# Mesoporous Silica vs. Organosilica Composites to Desulfurize Diesel

**DOI:** 10.3389/fchem.2019.00756

**Published:** 2019-11-14

**Authors:** Susana O. Ribeiro, Carlos M. Granadeiro, Marta C. Corvo, João Pires, José M. Campos-Martin, Baltazar de Castro, Salete S. Balula

**Affiliations:** ^1^LAQV-REQUIMTE, Departamento de Química e Bioquímica, Faculdade de Ciências, Universidade Do Porto, Porto, Portugal; ^2^CENIMAT/I3N, Departamento de Ciência dos Materiais, Faculdade de Ciências e Tecnologia, Universidade Nova de Lisboa, Caparica, Portugal; ^3^Faculdade de Ciências, Centro de Química e Bioquímica and CQE, Universidade de Lisboa, Lisbon, Portugal; ^4^Grupo de Energía y Química Sostenibles (EQS), Instituto de Catálisis y Petroleoquímica, CSIC, Madrid, Spain

**Keywords:** oxidative desulfurization, heterogeneous catalysis, mesoporous silica, polyoxometalate (POM), fuels

## Abstract

The monolacunary Keggin-type [PW_11_O_39_]^7−^ (PW_11_) heteropolyanion was immobilized on porous framework of mesoporous silicas, namely SBA-15 and an ethylene-bridged periodic mesoporous organosilica (PMOE). The supports were functionalized with a cationic group (*N*-trimethoxysilypropyl-*N, N, N*-trimethylammonium, TMA) for the successful anchoring of the anionic polyoxometalate. The PW_11_@TMA-SBA-15 and PW_11_@TMA-PMOE composites were evaluated as heterogeneous catalysts in the oxidative desulfurization of a model diesel. The PW_11_@TMA-SBA-15 catalyst showed a remarkable desulfurization performance by reaching ultra-low sulfur levels (<10 ppm) after only 60 min using either a biphasic extractive and catalytic oxidative desulfurization (ECODS) system (1:1 MeCN/diesel) or a solvent-free catalytic oxidative desulfurization (CODS) system. Furthermore, the mesoporous silica composite was able to be recycled for six consecutive cycles without any apparent loss of activity. The promising results have led to the application of the catalyst in the desulfurization of an untreated real diesel supplied by CEPSA (1,335 ppm S) using the biphasic system. The system has proved to be a highly efficient process by reaching desulfurization values higher than 90% for real diesel during three consecutive cycles.

## Introduction

Over the last years, ordered mesoporous silicas (OMS) have attracted researchers' attention in catalysis, due to its long range order, high surface areas and well-defined pore size (2–50 nm) (Esquivel et al., 2014; Wang et al., 2014). Moreover, the surface of these materials can be easily modified through reaction with organosilanes, incorporating proper functional groups on their surfaces that will guarantee the effective immobilization of appropriate compounds, such as active catalytic centers that will form novel heterogeneous catalysts.

Different mesoporous silica families (SBA-n, PMOs, etc.) have been used to create active heterogeneous catalysts, some of these having Keggin-type polyoxometalates (POMs) as active centers (Zhu et al., 2009). The morphology of these silica based materials and the functional groups present at their surface are crucial to the structural robustness of these catalysts.

Periodic mesoporous organosilicas (PMOs) are a recent class of ordered organic-inorganic hybrid mesoporous materials. PMOs allow the tuning of the superficial and physical properties besides being structurally robust (Esquivel et al., 2014; Park et al., 2014). Usually, the preparation of PMOs is conducted, in the presence of a structure-directing agent, by hydrolysis and condensation reactions of bridged silsesquioxane precursors with general formula (R′O)_3_-Si–R–Si–(OR′)_3_. The synthetic procedure for PMOs is similar to the preparation of mesoporous silicas, such as SBA-15 (Van Der Voort et al., 2013; Granadeiro et al., 2016). The functional organic moieties in PMOs are present at the surface but also in the channel walls, which are responsible for the structural features of the material (rigidity/flexibility) (Van Der Voort et al., 2013). The unique properties of PMOs make them suitable candidates for catalytic applications; however, the number of reports found in the literature is rather scarce and, in particular, its application in oxidative desulfurization is still inexistent (Karimi et al., 2012).

The demand for ultra-low sulfur fuels has motivated the development of new desulfurization methods, such as oxidative desulfurization (ODS), that brings technological, environmental, and economic advantages (Ribeiro et al., 2017; Yang et al., 2017; Liu et al., 2019). In ODS, the presence of an active catalyst with proper textural and surface chemical properties, as well as, an appropriate oxidant is the key to the success of this process. The monolacunary [PW_11_O_39_]^7−^Keggin polyoxometalate has shown remarkable performance in oxidative reactions (Casuscelli et al., 2004; Granadeiro et al., 2013; Ribeiro et al., 2013a; Singh et al., 2015; Coronel and da Silva, 2018), which has motivated its application in the oxidative desulfurization of fuels (Abdalla et al., 2009; Abdalla and Li, 2012; Wu et al., 2014; Mirante et al., 2018; Ribeiro et al., 2018b). In this work, novel composites have been prepared through the impregnation of the [PW_11_O_39_]^7−^ (PW_11_) heteropolyanion in silica materials functionalized with *N*-trimethoxysilypropyl-*N, N, N*-trimethylammonium (TMA). Two different mesoporous silica supports were selected to prepare novel composites: the ordered mesoporous silica SBA-15 and an ethylene-bridged (PMOE) periodic mesoporous organosilica. Both materials were tested in the oxidative desulfurization of simulant diesel under biphasic and solvent-free systems. In this work, a POM-based PMO (PW_11_@TMA-PMOE) was used for the first time in the oxidative desulfurization of model fuel. Moreover, the composite exhibiting better desulfurization performance (PW_11_@TMA-SBA-15) with the model fuel was also tested in the sulfur removal of an untreated diesel supplied by CEPSA, under biphasic and solvent-free systems.

## Experimental section

### Materials and Methods

The reagents used for the synthesis of the materials and the ODS experiments were purchased to chemical suppliers and used without further purification (Ribeiro et al., 2018b). The materials prepared were characterized as previously described by our group for other mesoporous silica-based composites (Ribeiro et al., 2017). The ODS studies with model diesel were followed by GC-FID in a previously described setup while for the experiments with real diesel the sulfur content was determined by ultraviolet fluorescence at Galp (Julião et al., 2017).

### Synthesis of PW_11_@TMA-SBA-15 Composite

The tetra-*n*-butylammonium [TBA, (C_4_H_9_)_4_N] salt of monolacunary phosphotungstate [PW_11_O_39_]^7−^ (PW_11_) was prepared according to the literature (Ribeiro et al., 2018b). The mesoporous silica SBA-15 functionalized with *N,N,N*-trimethylammonium groups (TMA-SBA-15) was initially prepared followed by the impregnation of PW_11_ according to previously reported methods (Mirante et al., 2017; Ribeiro et al., 2018a).

*TMA-SBA-15:* Anal. Found (%): N, 1.4; C, 7.6; H, 2.2; loading of TMA: 0.098 mmol per g; selected FT-IR (cm^−1^): 3,436 (vs), 2,360 (w), 1,868 (w), 1,635 (m), 1,489 (m), 1,479 (m), 1,419 (w), 1,086 (vs), 951 (m), 806 (s), 692 (w), 678 (w), 553 (w), 460 (s); selected FT-Raman (cm^−1^): 3,028 (vs), 2,981 (sh), 2,972 (vs), 2,931 (m), 2,925 (sh), 2,823 (m), 1,450 (s), 912 (m).

*PW*_11_@*TMA-SBA-15:* Anal. Found (%) W, 21.3; Si, 4.7; loading of POM: 0.099 mmol per g, Si/W (molar) = 1.46; selected FT-IR (cm^−1^): 3,444 (vs), 2,360(m), 2,341 (m), 1,644 (m), 1,447 (m), 1,195 (sh), 1,085 (vs), 948 (m), 914 (w), 856 (w), 809 (m), 458 (s); selected FT-Raman (cm^−1^): 3,035 (s), 2,979 (s), 2,922 (s), 2,902 (m), 1,444 (m), 976 (vs), 970 (vs).

### Synthesis of PW_11_@TMA-PMOE Composite

For the preparation of TMA-PMOE (Zhang et al., 2008; Granadeiro et al., 2016), Pluronic P123 (0.096 mmol) and KCl (47 mmol) were first dissolved in aqueous HCl (1.6 M; 29 mmol) at 40°C during 4 h. BTEE (2.84 mmol) and TMA (0.71 mmol) were added followed by stirring for 20 h at 40°C. The mixture was placed in an autoclave and heated at 100°C for 24 h. The solid was recovered by filtration and dried in a desiccator under silica gel. Finally, the solid was refluxed for 24 h in an acidic ethanol solution for the extraction of the co-polymer.

The PW_11_@TMA-PMOE composite was prepared by adding an acetonitrile solution of PW_11_ (1 g in 20 mL) to the TMA-PMOE (0.5 g) and allowing the mixture to stir at room temperature for 72 h. The solid was recovered by filtration, washed with acetonitrile and dried in a desiccator over silica gel.

*TMA-PMOE:* Anal. Found (%): N, 0.85; C, 19.79; H, 4.78; loading of TMA: 0.61 mmol per g; selected FT-IR (cm^−1^): 3,434 (vs), 2,886 (w), 2,360 (w), 1,637 (m), 1,488 (w), 1,413 (m), 1,270 (m), 1,159 (s), 1,093 (sh), 1,031 (vs), 912 (s), 763 (m), 696 (m), 441 (s); selected FT-Raman (cm^−1^): 2,972 (sh), 2,891 (vs), 2,802 (w), 1,441 (sh), 1,412 (m), 1,271 (m), 995 (m), 902 (w), 770 (m), 511 (s).

*PW*_11_@*TMA-PMOE:* Anal. Found (%) W, 10.24; Si, 1.55; loading of POM: 0.050 mmol per g, Si/W (molar) = 1.01; selected FT-IR (cm^−1^): 3,444 (vs), 2,975 (w), 2,898 (m), 2,360 (m), 2,343 (w) 1,652 (m), 1,488 (w), 1,417 (m), 1,270 (m), 1,159 (s), 1,079 (sh), 1,033 (vs), 908 (s), 815 (w), 767 (m), 696 (m), 501 (w), 441 (s); selected FT-Raman (cm^−1^): 3,031 (w), 2,968 (w), 2,891 (vs), 2,806 (w), 1,450 (m), 1,412 (s), 1,271 (m), 980 (s), 968 (sh), 928 (w), 760 (m), 515 (s).

### Oxidative Desulfurization Processes Using Model Diesel

The ODS experiments were performed using a multicomponent model diesel composed by 1-BT, DBT, 4-MDBT, and 4,6-DMDBT (500 ppm each) in *n*-octane. The ODS studies were performed using either a biphasic extractive and catalytic oxidative desulfurization (ECODS) or a solvent-free catalytic oxidative desulfurization (CODS) system. For the biphasic system, a 1:1 mixture of model diesel/MeCN (1.5 mL) was added to the heterogeneous composite (the amount corresponding to 3 μmol of POM), stirred for 10 min and an aliquot of the upper phase oil was taken. Afterwards, aqueous hydrogen peroxide 30% (40 μL; H_2_O_2_/S molar = 8) was added initiating the catalytic step. For the solvent-free studies, model diesel (750 μL), 3 μmol of active catalyst and H_2_O_2_ oxidant (H_2_O_2_/S molar = 4) were used. A final liquid-liquid extraction, was performed to remove the oxidized sulfur compounds, using an extraction solvent such as MeCN. The reactions were monitored by GC analysis using tetradecane as a standard. At the end of oxidation, centrifugation was carried out to separate the solid catalyst, which was washed with ethanol and dried in a desiccator over silica gel. At the end of each cycle, the catalyst was recovered and reused in a new ODS cycle under the same reactional conditions.

### Oxidative Desulfurization Process Using Untreated Diesel

An untreated diesel sample supplied by CEPSA (containing about 1,335 ppm of sulfur) was also desulfurized using the PW_11_@TMA-SBA-15 catalyst. The untreated diesel was mixed with the heterogeneous composite (an equivalent amount containing 3 μmol of PW_11_) in acetonitrile and with a H_2_O_2_/Sulfur ratio equal to 8. The mixture was heated at 70°C for 2 h, after which, the diesel was separated, washed with acetonitrile and separated by decantation. The CEPSA diesel was also used under solvent-free conditions by adding the catalyst and oxidant to diesel and heating at 70°C during 2 h. The mixture was centrifuged and the diesel washed with acetonitrile during 10 min. The catalyst was recycled for three cycles by recovering the catalyst, washing with ethanol and drying in a desiccator over silica gel overnight to be used in another ODS cycle.

## Results and Discussion

### Catalysts Characterization

**Scheme 1:**
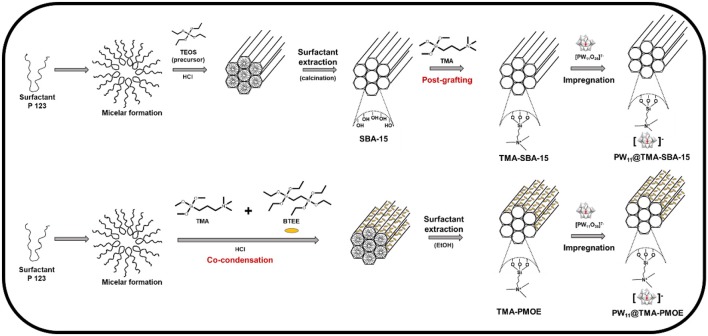
Representation of the synthetic pathway for the different PW_11_-based composites.

The synthesis of the TMA-functionalized silica supports and the preparation of the PW_11_-based composites is represented in Scheme 1. The synthesis of ethylene-bridged PMO has been performed by co-condensation of the bridged bis-silane [1,2-bis (triethoxysilyl) ethane; BTEE] and the terminal silane (TMA) in the presence of the micelles of the surfactant (Pluronic P123) in acid medium. In the end, an ethanol extraction is performed to remove the surfactant. The formation of SBA-15 is also based in the same surfactant using TEOS as silica precursor in acid medium. The surfactant is removed by calcination after an aging period. The functionalization of SBA-15 was performed *via* post-grafting with the same terminal silane (TMA; Wahab et al., 2005; Van Der Voort et al., 2013).

An impregnation method was used to immobilize the PW_11_ in TMA-functionalized silicas by electrostatic interactions. Several characterization techniques were used to assess the successful preparation of the materials.

**Figure 1:**
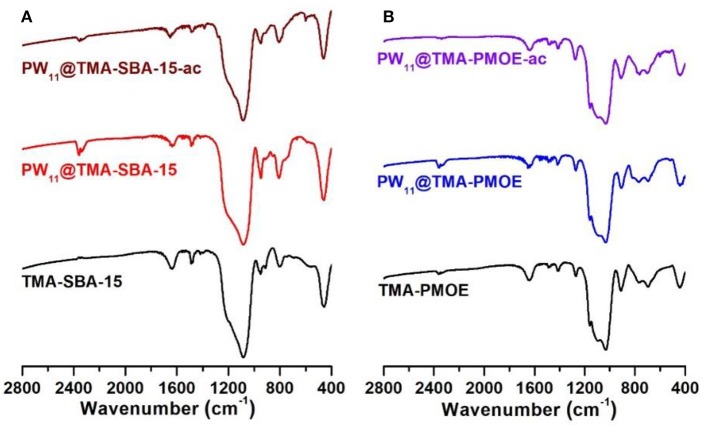
FT-IR spectra of **(A)** TMA-SBA-15 and **(B)** TMA-PMOE composites with PW_11_ before and after catalysis.

**Figure 2:**
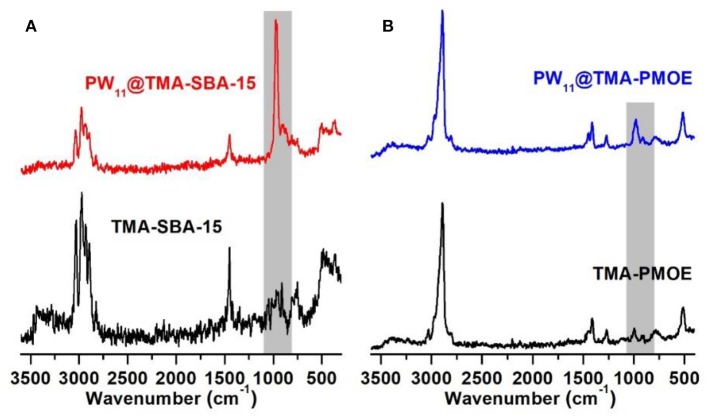
FT-Raman spectra of **(A)** TMA-SBA-15 and **(B)** TMA-PMOE composites with PW_11_.

**Figure 3:**
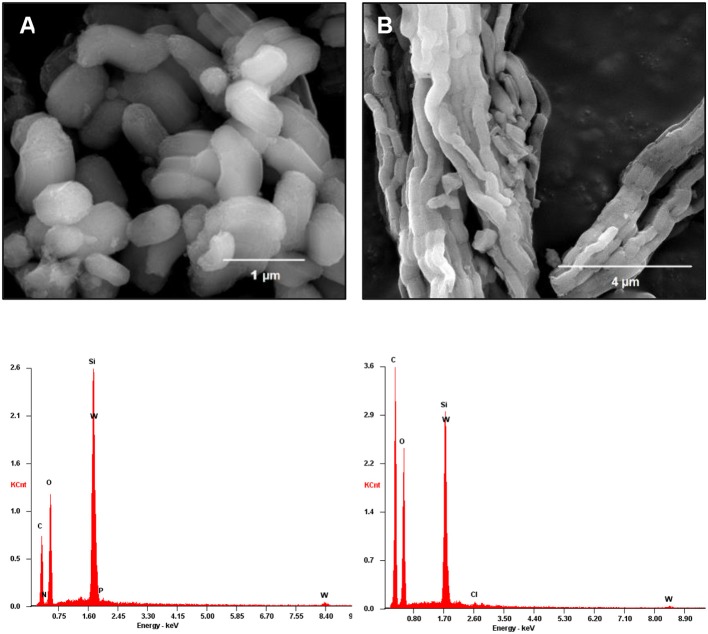
SEM images and EDS spectra of the PW_11_ composites: **(A)** PW_11_@TMA-SBA-15 and **(B)** PW_11_@TMA-PMOE.

The FT-IR spectra of the prepared TMA-functionalized supports and the resulting PW_11_ composites are displayed in Figure 1. The spectrum of PW_11_@TMA-SBA-15 presents a similar profile to that of TMA-SBA-15. The typical bands assigned to the siliceous support located at 1,100-400 cm^−1^ range namely the ν_as_(Si–O–Si), ν_s_(Si–O–Si), and δ(O–Si–O) vibrational modes, respectively (Pires et al., 2016; Mirante et al., 2017; Ribeiro et al., 2017, 2018a), mask the bands that could be assigned to the POM incorporation and no extra bands can be recognized. Nevertheless, the intense bands observed in the 1,010–860 cm^−1^ range of the FT-Raman spectrum (Figure 2A) confirm the presence of the POM in the composite. The bands associated with the vibrational modes of the amine groups can also be observed, in particular, in the 3,035–2,902 and 1,450–1,412 cm^−1^ ranges for ν(C-H) and δ(CH_2_), respectively. The presence of POM in this composite material was also confirmed by the presence of W in the EDS spectrum (Figure 3A) and elemental analysis with a loading of PW_11_ of 0.099 mmol per g of material.

The FT-IR spectrum of the TMA-PMOE material (Figure 1B) presents the typical bands of silsesquioxane frameworks namely the intense band in at 1,093 cm^−1^ assigned to ν_as_(Si–O–Si), as well as the bands located at 912, 763, and 441 cm^−1^ associated with the ν_as_(Si–OH), ν_s_(Si–O–Si), and δ(Si–O–Si), respectively (Zhu et al., 2002; Zhang et al., 2005, 2008; Granadeiro et al., 2016). The band at 2,898 cm^−1^ (stretching) and at 1,413 cm^−1^ (bending) can be associated to the C–H vibrational modes of bridging-ethylene and the alkyl groups of TMA (Zhang et al., 2005, 2008; Granadeiro et al., 2016). The absence of bands located around 1,340 and 1,380 cm^−1^ suggest that the Pluronic P123 surfactant has been successfully removed during the extraction process (Li et al., 2007). The FT-IR spectrum of the PW_11_@TMA-PMOE composite suggests that the structure of the support, previously described, has been maintained. The presence of PW_11_ in the composite material is not evident in the FT-IR spectrum, due to the presence of intense bands arising from TMA-PMOE. As previously discussed, FT-Raman allows a better identification of the POM vibrational bands due to the lower intensity of the bands from the siliceous supports (Zhang and Yang, 2008; Abdalla and Li, 2012; Julião et al., 2015).

The FT-Raman spectrum of PW_11_@TMA-PMOE (Figure 2B) is mainly dominated by the bands from PMOE, in particular, the bands in the 3,000–2,800 cm^−1^ range ascribed to ν(C-H), at 1,412 and 1,271 cm^−1^ assigned to the twisting and wagging modes of CH_2_, respectively, and the band at 515 cm^−1^ assigned to the vibration of the ethylene unit against the siliceous framework together with δ(Si–O–Si) vibrations (Hoffmann et al., 2007; Granadeiro et al., 2016). The presence of PW_11_ is suggested by the band at 980 cm^−1^ which can be attributed to the ν_as_(W-O_d_) vibrations (Ribeiro et al., 2018b). Further suggest the presence of PW_11_ in the composite material was confirmed by EDS (Figure 3B) with the presence of W in the EDS spectrum, as well as by elemental analysis of W with a loading of 0.057 mmol of POM per 1 g of material.

**Figure 4:**
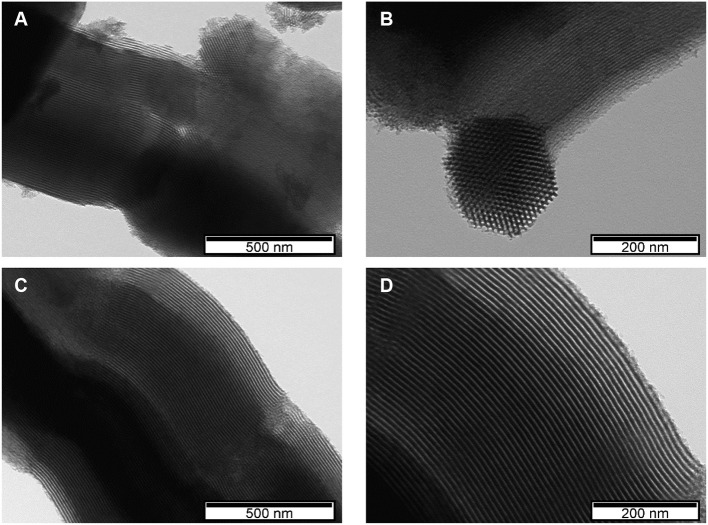
TEM images of the **(A,B)** PW_11_@TMA-SBA-15 and **(C,D)** PW_11_@TMA-PMOE composites at different magnifications.

The SEM image of the TMA-SBA-15 support (Figure S1) presents the typical hexagonal elongated particles of the mesoporous SBA-15 framework. The SEM image of its analog composite (Figure 3A) reveals that the morphology of TMA-SBA-15 was retained after the POM incorporation. The SEM image of PW_11_@TMA-PMOE (Figure 3B) still exhibits the same morphology as the support (Figure S1) composed by ropelike structures with several micrometers in length (Inagaki et al., 1999; Granadeiro et al., 2016). The chemical composition of the composites was evaluated by EDS. The results reveal, besides silicon as the main element, the presence of tungsten which is consistent with the incorporation of PW_11_ on the final composites (Figure 3). TEM analysis of both PW_11_-composites were also performed for a more detailed observation of the morphology (Figure 4). The images show highly ordered and uniform pores organized in long channels as well as the typical honeycomb-shaped hexagonal mesopores. The results obtained from electronic microscopy strongly suggest the structural preservation of the supports in the final composites.

**Figure 5:**
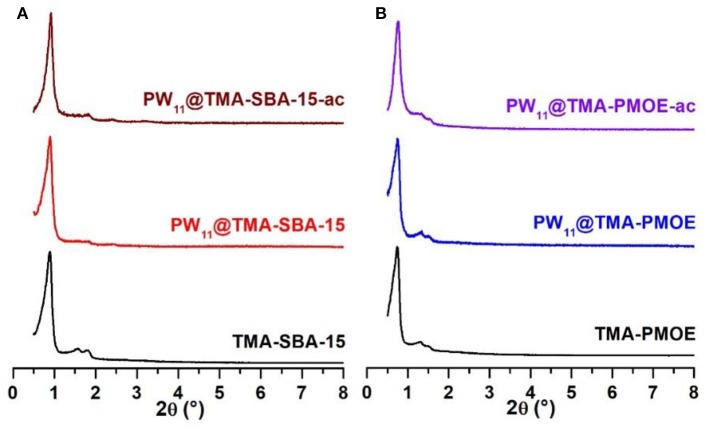
Powder XRD patterns of **(A)** TMA-SBA-15 and **(B)** TMA-PMOE composites with PW_11_ (ac stands for after catalysis).

The powder XRD patterns of the TMA-functionalized supports and the resulting PW_11_ composites are presented in Figure 4. The powder XRD of the PW_11_@TMA-SBA-15 composite (Figure 5A) exhibit the typical low-angle three peaks of SBA-15 materials, with a shift to higher 2θ and with lower intensity for the (110) and (200) reflections, as previously reported in other POM-incorporated SBA-15 composites (Zhao et al., 1998; Kruk et al., 2000; Ribeiro et al., 2013b). The absence of peaks from the PW_11_ points out that the POM has been successfully incorporated. In the case of the mesoporous organosilicas support (TMA-PMOE), the pattern also exhibits the same three peaks as seen in the SBA-15 materials. The highly ordered mesostructure of the prepared PMO was accomplished by the addition of a KCl as additive which improved the interaction between the organosilica oligomers and the surfactant (Zhang et al., 2008; Granadeiro et al., 2016). The powder XRD pattern of the PW_11_@TMA-PMOE composite is similar to the TMA-PMOE support, indicating structural preservation of the support.

**Figure 6:**
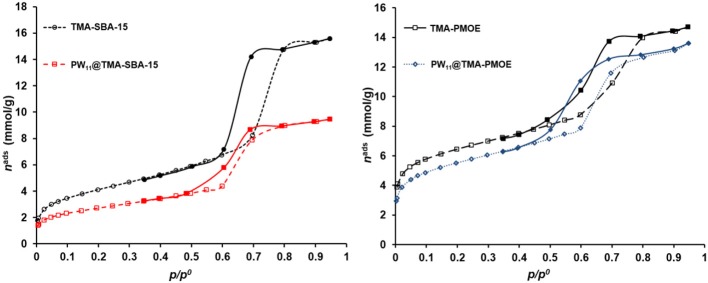
N_2_ adsorption-desorption isotherms of the TMA-SBA-15 and PW_11_@TMA-SBA-15 composite **(left)**; TMA-PMOE and PW_11_@TMA-PMOE **(right)**.

The textural properties of SBA-15 and ethylene-bridge PMO materials were evaluated by N_2_ adsorption experiments. Both materials exhibited type IV isotherms with H1 hysteresis loops typical of mesoporous materials (Figure 6). The results obtained for the starting supports and the PW_11_-composites are summarized in Table 1. The S_BET_ and V_p_ values obtained for both composites are smaller when compared with the corresponding support, which confirms the incorporation of POM inside the pore channels (Granadeiro et al., 2016; Mirante et al., 2017; Ribeiro et al., 2018b).

**Table 1:** Textural parameters of the TMA-functionalized supports and the resulting PW_11_ composites.

**Material**	**S_**BET**_ (m^**2**^.g^**−1**^)**	**V_**p**_ (cm^**3**^.g^**−1**^)**
TMA-SBA-15	336	0.54
PW_11_@TMA-SBA-15	221	0.32
TMA-PMOE	521	0.51
PW_11_@TMA-PMOE	449	0.47

**Figure 7:**
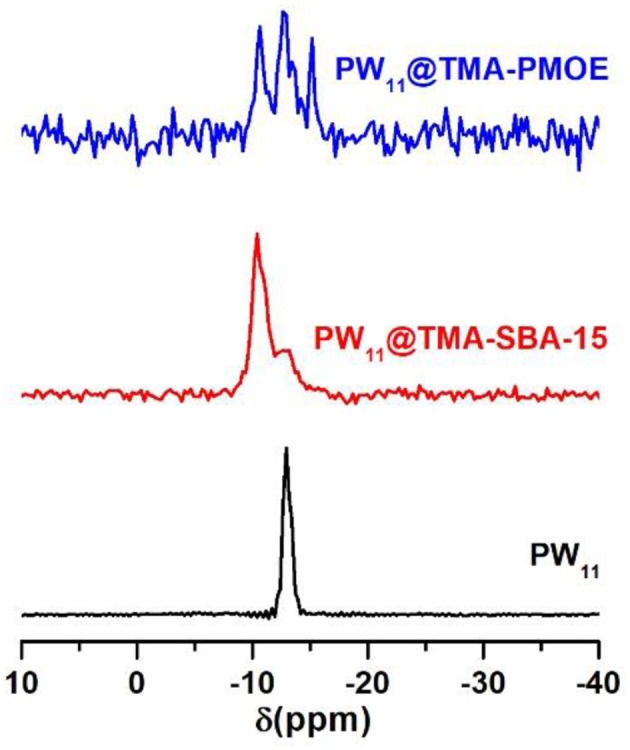
^31^P MAS NMR spectra of PW_11_, and the corresponding PW_11_@TMA-SBA-15 and PW_11_@TMA-PMOE composites.

The composites were also studied by ^31^P MAS-NMR (Figure 7) in order to check the structure of the POM inside the silica materials. The spectrum of the PW_11_@TMA-SBA-15 composite presents a broad peak centered at −10.41 ppm with a prominent shoulder at −12.75 ppm. The shoulder corresponds to free PW_11_ anion while the broad peak may be resultant from the interaction of the POM with the siliceous matrix. In fact, a downfield shift in the ^31^P NMR signal of POMs has been reported as a result of the interaction with Si-OH^2+^ groups of the silica support (Morales et al., 2018). This result indicates that the PW_11_ structure was retained after its incorporation in the TMA-SBA-15 support. The spectrum of PW_11_@TMA-PMOE presents three different peaks with approximately similar intensities at −10.64, −12.79, and −15.16 ppm. The two peaks located at −10.64 and −12.79 ppm should correspond to the free PW_11_ and PW_11_ interacting with the support, respectively, as previously discussed. The peak at −15.16 ppm should correspond to the PW_11_ anion with occupied lacuna which is known to promote an upfield shift of the ^31^P signal (Guo et al., 2002; Granadeiro et al., 2013).

**Figure 8:**
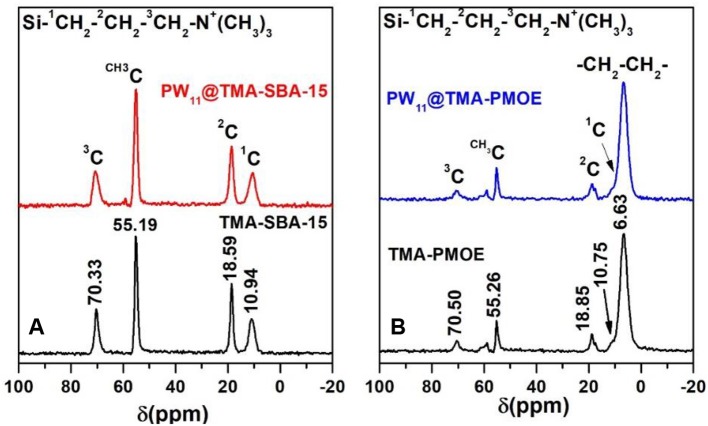
^13^C CP MAS NMR spectra of the TMA-functionalized supports and the resulting PW_11_ composites.

The TMA-functionalized SBA-15 and ethylene-bridged PMO supports as well as the resulting PW_11_ composites were analyzed by ^13^C CP MAS NMR spectroscopy (Figure 8). The spectrum of the TMA-SBA-15 support exhibited four peaks located at 70.33, 55.19, 18.59, and 10.94 ppm (Figure 8A). The peak located at 55.19 ppm correspond to the methyl group and the others can be assigned to the C3 (70.33), C2 (18.59), and C1(10.94) carbon atoms of the TMA group, respectively, Si-^1^CH_2_-^2^CH_2_-^3^CH_2_-N^+^(CH_3_)_3_. The nonexistence of ^13^C signals in the 67–77 ppm range gives a positive indication that the surfactant has been efficiently removed (Hoffmann et al., 2007). The spectrum of the PW_11_@TMA-SBA-15 also presents chemical shifts similar to those of the support material, namely at 70.74, 55.16, 18.61, and 10.53 ppm.

The spectrum of TMA-PMOE exhibits an intense peak at 6.63 ppm corresponding to the –CH_2_-CH_2_- groups (Figure 8B), and three peaks at 10.75, 18.85, and 70.50 ppm assigned to the C1, C2, and C3 of TMA, respectively. The spectrum, also presents a peak at 61.37 ppm ascribable to CH_3_-CH_2_-O groups most likely due to incomplete hydrolysis of 1,2-bis (triethoxysilyl) ethane or during the ethanolic extraction (Zhang et al., 2005, 2008; Granadeiro et al., 2016).

**Figure 9:**
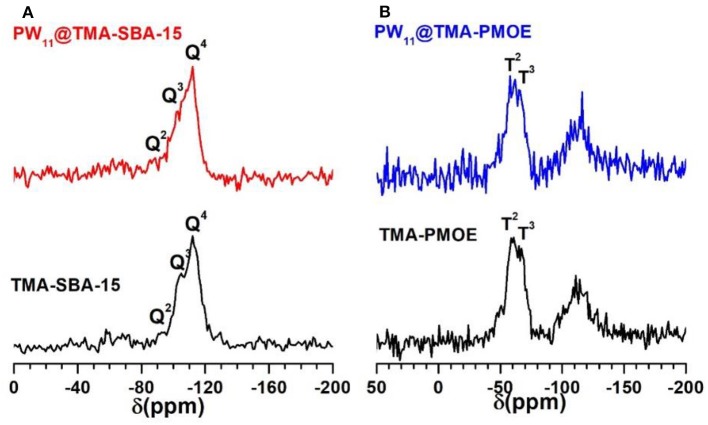
^29^Si MAS NMR of **(A)** TMA-SBA-15 and **(B)** TMA-PMOE composites with PW_11_.

The ^29^Si MAS NMR solid state spectrum of the TMA-SBA-15 support presents a broad band at δ ≈ −112 ppm corresponding to Q^4^ species as well as two shoulders at δ ≈ −105 ppm and −93 ppm assigned to Q^3^ and Q^2^ species, where Q^n^ = Si(OSi)_4−n_(OH)_n_, *n* = 2–4 (Figure 9A) (Zhang and Yang, 2008; Mauder et al., 2009; Ribeiro et al., 2018b). An identical profile could be observed in the spectrum of PW_11_@TMA-SBA-15 suggesting that the siliceous framework was preserved in the final composite. The ^29^Si MAS spectrum of the ethylene-bridge PMO (Figure 9B) presents the characteristic T^n^ signals attributed to [C–Si(OSi)_2_(OH)] (T^2^ at −60.8 ppm) and [C–Si(OSi)_3_] (T^3^ at −67.1 ppm) and some Q^n^ signals (Si sites attached to four oxygen atom) between −100 and −115 ppm. This is indicative of some cleavage of the Si-C bond during the synthesis and surfactant extraction process (Granadeiro et al., 2016; Huang et al., 2017). The incorporation of PW_11_ in the TMA-PMOE support did not result in significant changes in the ^29^Si MAS spectrum of the composite when compared to the starting support material, which also indicates the preservation of the siliceous structure.

### Oxidative Desulfurization Processes Using Model Diesel

A model diesel with ~2,000 ppm S was used for the desulfurization studies which were carried out at 70°C. The PW_11_@TMA-SBA-15 composite and the PW_11_@TMA-PMOE composite were tested as heterogeneous catalysts. The oxidative desulfurization of the model diesel was accomplished using ether a biphasic ECODS system or a solvent-free CODS system. For both systems, the equivalent amount of composite containing 3 μmol of PW_11_ were used.

An initial liquid-liquid extraction was performed (10 min of stirring at 70°C) for the biphasic system in the presence of the catalyst. During this step, some sulfur-containing compounds were removed from the model oil to the solvent phase, until the transfer equilibrium is reached. Afterwards, the oxidative catalytic stage was initiated by adding the oxidant (ratio H_2_O_2_/S = 8, at 70°C). In this step, the sulfur compounds were simultaneously oxidized and extracted to the MeCN phase. The solvent-free system, begins with the catalytic stage, in the absence of extraction solvent (ratio H_2_O_2_/S = 4, at 70°C), followed by a final extraction step with MeCN or water to remove the oxidized sulfur compounds.

**Figure 10:**
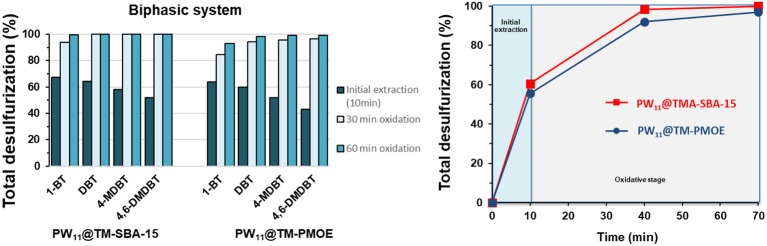
Individual desulfurization percentages **(left)** and total desulfurization **(right)** using the biphasic system (1:1 model diesel/MeCN; H_2_O_2_/S = 8; 70°C), using the PW_11_@TMA-SBA-15 and PW_11_@TMA-PMOE catalysts.

Figure 10 displays the results obtained for the biphasic system using both heterogeneous catalysts: PW_11_@TMA-SBA-15 and PW_11_@TMA-PMOE. It can be observed that during the initial extraction step (10 min stirring) between 55 and 60% of the sulfur-containing compounds are transferred into the MeCN phase. This transference follows the order 1-BT > DBT > 4-MDBT > 4,6-DMDBT, with 1-BT being the most easily extracted, which can be justified by the size and geometry of each compound (Ribeiro et al., 2013a, 2016). In the oxidative catalytic step, the non-oxidized sulfur compounds, mostly present in the solvent phase, are oxidized and simultaneously more sulfur compounds are transferred to the solvent phase (Julião et al., 2015). The PW_11_@TMA-SBA-15 catalyst was able to achieve complete desulfurization for DBT, 4-MDBT and 4,6-DMDBT and 93.9% for 1-BT, after 30 min of the catalytic step initiation. After 60 min, only 2 ppm of 1-BT remained in the model diesel. The oxidative reactivity follows the expected order, with 1-BT being the most difficult compound to be oxidized, as a result of steric hindrance and the different electronic densities of the sulfur atoms (Ribeiro et al., 2013a, 2018b; Julião et al., 2015; Mirante et al., 2017). Regarding the PW_11_@TMA-PMOE catalyst, the desulfurization efficiency was slightly lower reaching after 60 min of catalytic oxidation, 92.8% for 1-BT, 98.2% for DBT, 99.0% for 4-MDBT and 99.3% for 4,6-DMDBT, resulting in a total desulfurization of 96.9%.

**Figure 11:**
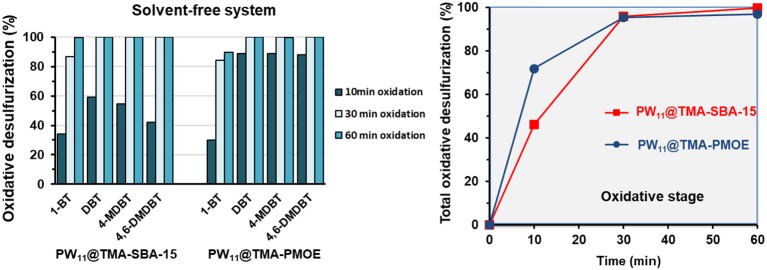
Individual desulfurization percentages **(left)** and total oxidative desulfurization **(right)**, using the solvent-free system (ratio H_2_O_2_/S = 4 at 70°C) and PW_11_@TMA-SBA-15 and PW_11_@TMA-PMOE as catalysts (containing 3 μmol of PW_11_ active center).

The desulfurization results for the solvent-free system using both composites are presented in Figure 11. During the initial 10 min of catalytic oxidation, the PW_11_@TMA-PMOE catalyst is faster in the oxidation of sulfur compounds, achieving 72.0% of total oxidation, while the PW_11_@TMA-SBA-15 reached 45.3%. This might be related to the higher hydrophobicity of the PW_11_@TMA-PMOE composite that possesses more affinity with the diesel phase than PW_11_@TMA-SBA-15. Nevertheless, after 30 min of the process, the performance of both catalysts is similar, achieving total conversion for DBT, 4-MDBT and 4,6-DMDBT, and 84.2 and 86.8% conversion for 1-BT, using PW_11_@TMA-PMOE and PW_11_@TMA-SBA-15, respectively. At the end of 60 min of desulfurization, the SBA-15 composite reached ultra-low levels of sulfur with only 2 ppm of 1-BT (99.6% conversion) remaining in the model diesel, while with the PMOE composite only 89.6% of 1-BT has been converted (52 ppm remaining). In fact, even continuing the reaction up to 120 min, the conversion of 1-BT is still 93.8% using the PW_11_@TMA-PMOE. It seems that, despite the initial fast reaction rate (first 30 min), desulfurization gradually becomes slower and total conversion could not be reached even after 120 min. A similar behavior was also observed with the biphasic system since total desulfurization only reached 98.1 and 98.3%, after 120 and 240 min reaction, respectively.

**Figure 12:**
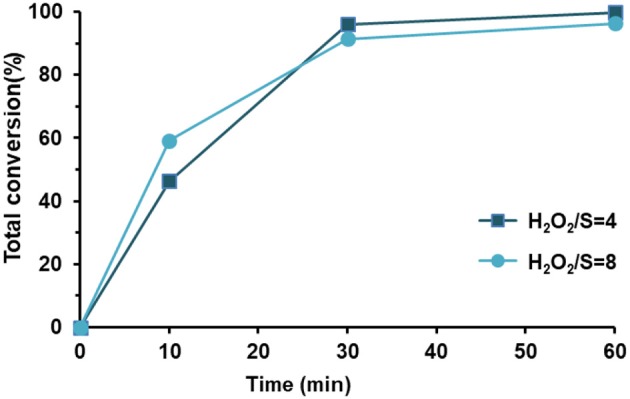
Total conversion for sulfur oxidation presented in the model diesel, using the solvent-free system at 70°C and PW_11_@TMA-SBA-15 catalyst in the presence of two different H_2_O_2_/S ratios.

Increasing the amount of oxidant in the solvent-free system to a ratio of H_2_O_2_/S = 8, ultra-low levels of sulfur could be achieved (7 ppm) with PW_11_@TMA-PMOE at the end of 60 min. Contrastingly, increasing the amount of oxidant (H_2_O_2_/S = 4 to H_2_O_2_/S = 8) the desulfurization efficiency of PW_11_@TMA-SBA-15 shows a slight decrease (Figure 12). In summary, the PW_11_@TMA-PMOE needs a higher amount of oxidant than the SBA-15 composites, which exhibits better desulfurization efficiencies with a H_2_O_2_/S = 4 ratio than H_2_O_2_/S = 8 (Ribeiro et al., 2018b).

### Recyclability of PW_11_@TMA-SBA-15

**Figure 13:**
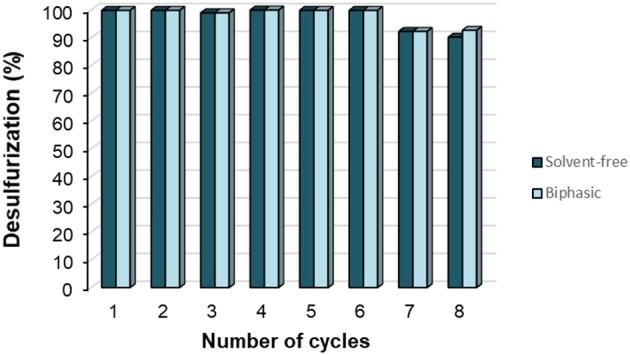
Recycling studies with PW_11_@TMA-SBA-15 composite after 60 min of the oxidative step under the solvent-free (H_2_O_2_/S = 4) and biphasic (H_2_O_2_/S = 8) systems at 70°C.

The recycling ability of PW_11_@TMA-SBA-15 was evaluated for several consecutive cycles using both desulfurization systems (biphasic and solvent-free). The desulfurization percentages obtained for eight cycles with the PW_11_@TMA-SBA-15 catalyst after 60 min of oxidation using the biphasic or the solvent-free systems are displayed in Figure 13. The results show that both systems maintain the desulfurization performance along six consecutive cycles. After the sixth cycle, some loss of catalytic activity was detected probably due to the active site deactivation by the presence of sulfones strongly adsorbed in the catalyst surface (Mirante et al., 2017, 2018).

### Catalysts Stability

The recovered PW_11_ composites were studied by several characterization techniques to access their robustness after catalytic use. The ICP-OES of the PW_11_@TMA-SBA-15 composite suggest that pratically no leaching of PW_11_ occurs using the solvent-free system, since the Si/W (molar) ratios before (1.46) and after catalysis (1.44 after eight desulfurization cycles) are very similar. The analysis performed after one desulfurization cycle, using the biphasic system, detected some leaching of the PW_11_ from the support material shown by the increase in the Si/W (molar) ratio from 1.46 to 1.79 after catalytic use. The ICP results for PW_11_@TMA-PMOE revealed that, after its use in the solvent-free system, the Si/W ratio was maintained (1.01 before and 1.00 after catalytic use).

The powder XRD patterns of PW_11_@TMA-SBA-15-ac and PW_11_@TMA-PMOE-ac after use in the biphasic system, exhibit similar profiles to the patterns of the as-prepared materials (Figure 5), which shows that both composites are robust and stable catalysts maintaining the structure of the siliceous support.

Regarding FT-IR spectra, the main vibrational bands of the composites after catalytic use remain practically unchanged, which is consistent with its structural retention (Figure 1). The morphology of the composites also seems to have been preserved after the desulfurization process as observed by SEM and TEM (Figures S2, S3), while the corresponding EDS spectra of each composite confirm the presence of PW_11_ by still exhibiting tungsten. Moreover, the EDS spectra of the PW_11_@TMA-SBA-15-ac also reveals the presence of S, resulting from oxidized sulfur-containing compounds from the model diesel that precipitated in the presence of the composite (Figure S2).

The ^31^P MAS NMR spectra of the composites before and after catalysis, using both desulfurization systems, have also been compared (Figure S4). The spectrum of PW_11_@TMA-SBA-15 after a biphasic cycle presents a broad peak at −12.68 ppm with a shoulder at −10.92 ppm. The results means that, after the biphasic cycle, the predominant species in the material is now the PW_11_ interacting with the support rather than the free PW_11_ as in the as-prepared composite. After eight cycles under the solvent-free system, the spectrum displays a main peak at −15.19 ppm that can be assigned to the PW_11_ with a saturated lacuna, probably by sulfone or peroxo interactions.

The PW_11_@TMA-PMOE after one biphasic desulfurization cycle exhibits a broad single peak centered at −10.72 ppm, while after one solvent-free cycle, an additional peak is also observed at −12.27 ppm. These peaks correspond to free PW_11_ (−10.64 ppm) and PW_11_ interacting with the silica support (−12.27 ppm), showing the structural retention of these species from the as-prepared material.

### Oxidative Desulfurization Process Using Untreated Diesel

**Figure 14:**
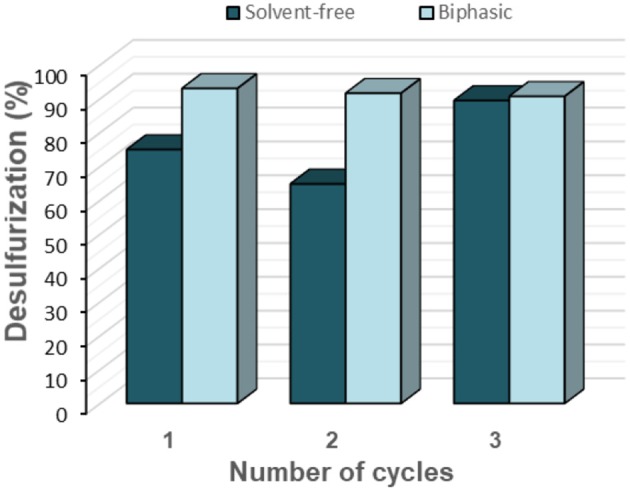
Desulfurization results of a real untreated diesel obtained after 2 h, catalyzed by PW_11_@TMA-SBA-15 at 70°C, using the solvent-free biphasic systems with H_2_O_2_/S = 8.

The PW_11_@TMA-SBA-15 was selected for application in the desulfurization of an untreated diesel supplied by CEPSA (1335 ppm S). Figure S5 presents the GC-FID/SCD analysis of this diesel. These results reveal that dibenzothiophenes and dibenzothiophene derivatives are the main sulfur compounds present in this untreated diesel. These studies were performed using the biphasic and also the solvent-free conditions at 70°C with H_2_O_2_/S = 8 during 2 h of oxidation. After the oxidative catalytic process, a liquid-liquid extraction with 1:1 MeCN/diesel at room temperature was performed for all treated diesel samples, during 10 min with stirring. At the end of the first cycle, the best performance was obtained with the biphasic system (93.1%), performing an initial extraction (1:1 diesel/MeCN) before oxidation step and also a final extraction after oxidation (Figure 14). On the other hand, under the solvent-free system, a lower desulfurization efficiency was reached (75%).

The solid PW_11_@TMA-SBA-15 catalyst was further recycled for two more consecutive cycles following the previously described procedure for simulant diesel. The biphasic system showed higher recycling ability than the solvent-free system, since catalyst efficiency is maintained for three consecutive cycles. On the other hand, an increase of desulfurization efficiency is observed from the second to the third cycle in the solvent-free system. This must be related to the formation of catalytic active intermediates during the previous desulfurization cycles. These active intermediates can be attributed to active peroxo compounds.

In summary, the PW_11_@TMA-SBA-15 using the biphasic system revealed to be a promising process for the desulfurization of untreated diesel reaching a desulfurization of 93.1% from CEPSA diesel. Moreover, the catalyst maintained its catalytic activity over three consecutive desulfurization cycles.

## Conclusion

In this work, two different POM-based silica composites were prepared via impregnation of PW_11_ on TMA-functionalized mesoporous silica (SBA-15 and ethylene-bridged PMO). The cationic functional group promotes the immobilization of the anionic PW_11_ by electrostatic interaction. The surface modification of SBA-15 was accomplished by post-synthetic grafting, while the introduction of functional groups in PMO was achieved *in situ* (“co-condensation”).

The desulfurization performance of PW_11_@TMA-SBA-15 and PW_11_@TMA-PMOE composites was evaluated using a multicomponent model diesel. The PW_11_@TMA-PMOE composite achieved 96.9% of desulfurization after 60 min of oxidative reaction, while the PW_11_@TMA-SBA-15 allowed to reach ultra-low levels of sulfur (<10 ppm) under the same period of time, using the biphasic system.

Complete conversions of DBT, 4-MDBT, and 4,6-DMDBT could be achieved for both catalysts after only 30 min under the solvent-free system. The PW_11_@TMA-PMOE catalyst revealed to be slightly less active than the PW_11_@TMA-SBA-15 catalyst, since the complete desulfurization of 1-BT could not be reached, after 60 min of the process. In comparison, the PW_11_@TMA-SBA-15 reached ultra-low levels of sulfur (2 ppm) for the same period of time. Moreover, the PW_11_@TMA-SBA-15 has shown a remarkable recycling ability, in both desulfurization systems, by maintaining its catalytic efficiency for six consecutive cycles.

The robustness of the composites was confirmed by characterization studies of the recovered solid catalysts suggesting their structural and chemical preservation after catalytic use.

The remarkable performance of PW_11_@TMA-SBA-15 with simulant diesel has led to its application with an untreated diesel, under biphasic and solvent-free systems. Furthermore, recycling tests were also performed using both systems for three consecutive cycles. The best result was obtained using the biphasic system removing 93.1% of sulfur compounds from the diesel after only 2 h and maintaining its remarkable desulfurization efficiency for two additional cycles. These successful recycling studies using a real untreated diesel, strongly indicates that the PW_11_@TMA-SBA-15 catalyst under biphasic conditions can be a promising system for reaching sulfur-free fuels.

## Data Availability Statement

All datasets generated for this study are included in the article/Supplementary Material.

## Author Contributions

SR (supervised by BC and SB) performed all the experimental work related with the preparation of the materials and the oxidative desulfurization reactions. CG contributed with the discussion of the characterization data and manuscript preparation. MC conducted the MAS NMR experiments. JP performed the textural characterization of the samples. JC-M co-supervised the desulfurization experiments with real diesel.

### Conflict of Interest

The authors declare that the research was conducted in the absence of any commercial or financial relationships that could be construed as a potential conflict of interest.
