# Supplementary material for: Mesoporous Silica vs. Organosilica Composites to Desulfurize Diesel
Source: Front Chem. 2019 Nov 14;7:756. doi: 10.3389/fchem.2019.00756 (PMC6868090; doi:10.3389/fchem.2019.00756)
Supplement: Supplementary file 1 [file Data_Sheet_1.docx]

ELECTRONIC SUPPORTING INFORMATION

**Catalytic oxidative desulfurization performance of mesoporous silica versus organosilica composites to treat model and real diesel**

Susana O. Ribeiro,^a^ Carlos Granadeiro,^a^ Marta C. Corvo,^b^ João Pires,^c^ José M. Campos-Martin,^d^ Baltazar de Castro^a^ and Salete S. Balula^a*^

*^a^ LAQV-REQUIMTE, Departamento de Química e Bioquímica, Faculdade de Ciências, Universidade do Porto, 4169-007 Porto, Portugal*

*^b^ CENIMAT/I3N, Faculdade de Ciências e Tecnologia, Universidade Nova de Lisboa, 2829-516 Monte da Caparica, Portugal*

*^c^ Centro de Química e Bioquímica and CQE, Faculdade de Ciências, Universidade de Lisboa, 1749-016 Lisboa, Portugal*

*^d^ Grupo de Energía y Química Sostenibles (EQS), Instituto de Catálisis y Petroleoquímica, CSIC, Marie Curie, 2 Canto Blanco, Madrid, Spain.*


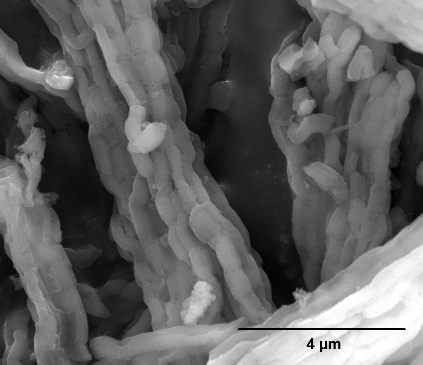

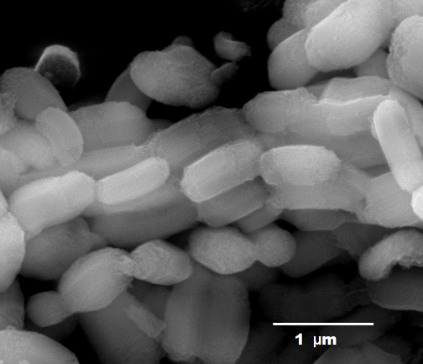


B

A

**Figure S1.** SEM images of the TMA-functionalized supports: (A) TMA-SBA-15 and (B) TMA-PMOE.


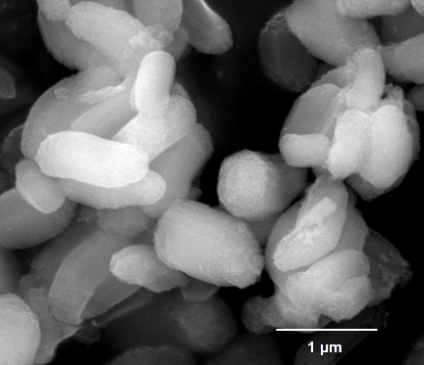


A


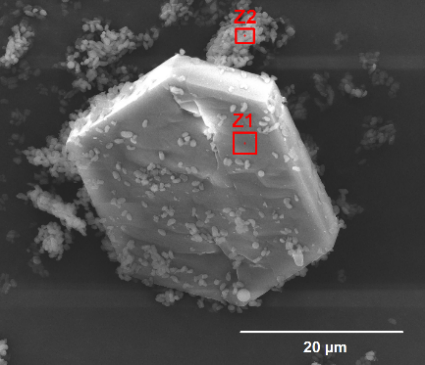


A


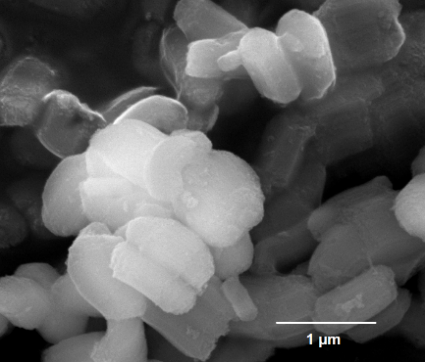


B


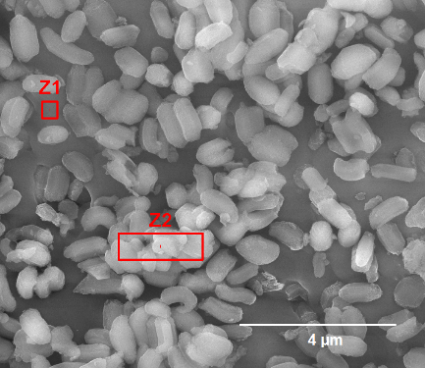


B


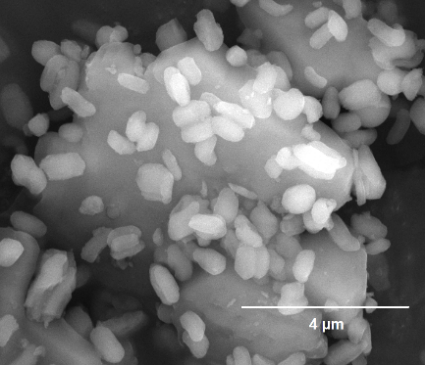


C


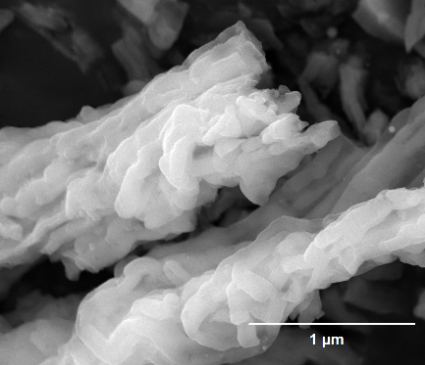


D


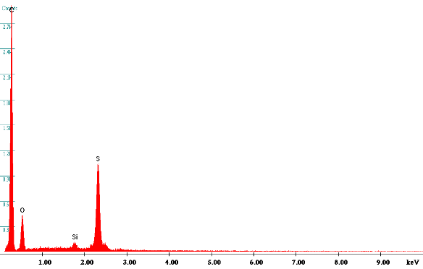


A - Z1


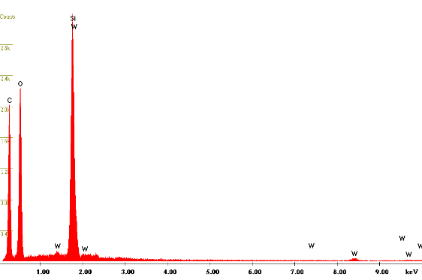


A – Z2


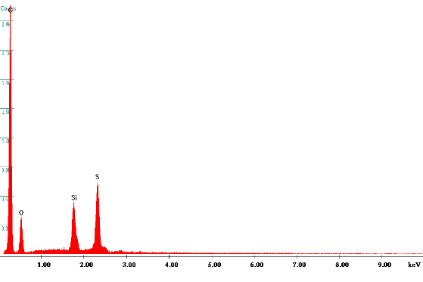


B – Z1


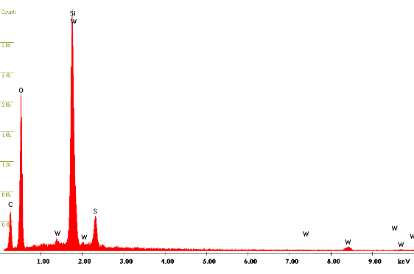


B – Z2


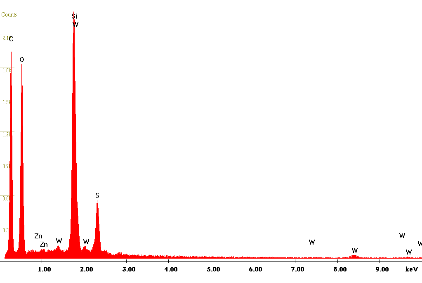


C


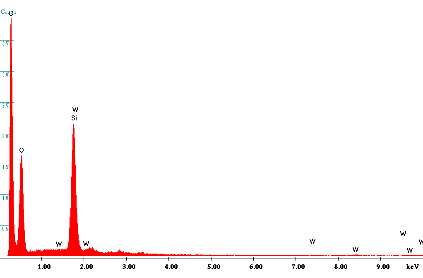


D

**Figure S2.** SEM images and EDS spectra of A - PW_11_@TMA-SBA-15 composite after one cycle using the biphasic system; B - PW_11_@TMA-SBA-15 composite after one cycle using the solvent-free system; C - PW_11_@TMA-SBA-15 composite after eight cycles using the solvent free system and D - PW_11_@TMA-PMOE composite after catalytic use using the solvent-free system.







**A**

**B**







**C**

**D**

**Figure S3.** TEM images of the PW_11_@TMA-SBA-15 (A,B) and PW_11_@TMA-PMOE (C,D) composites after catalytic use under the solvent-free and biphasic systems, respectively.


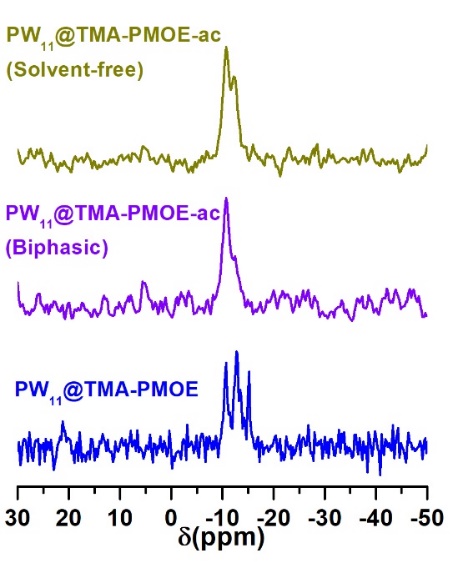

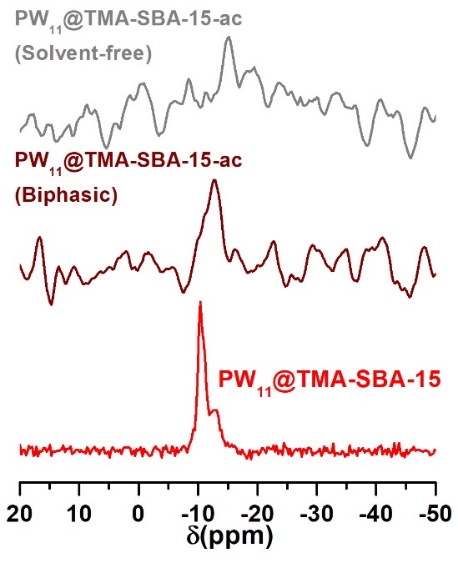


**Figure S4.** ^31^P MAS NMR spectra of the PW_11_@TMA-SBA-15 composite (left) and PW_11_@TMA-PMOE (right) before and after catalytic use (ac stands for after catalysis).

**Figure S5**. Chromatogram obtained by GC-FID/SCD from untreated diesel supplied by CEPSA (A) and model diesel with the identification of various sulfur compounds (B).
